# Effect of Asymmetric Fins on Thermal Performance of Phase Change Material-Based Thermal Energy Storage Unit

**DOI:** 10.3390/ma16072567

**Published:** 2023-03-23

**Authors:** Muhammad Shaban, Talha Irfan Khan, Muhammad Anwar, Meshal Alzaid, Rakan Alanazi

**Affiliations:** 1 Department of Mechanical Engineering, Institute of Space Technology, Islamabad P.O. Box 2750, Pakistan; m.shabansaify@gmail.com (M.S.); tkhan13@ku.edu.tr (T.I.K.); manwar18@gmail.com (M.A.); 2 Department of Physics, College of Science, Jouf University, Al-Jouf, Sakaka P.O. Box 2014, Saudi Arabia; rakan.ksa1418@hotmail.com

**Keywords:** phase change materials (PCMs), thermal energy storage unit (TESU)

## Abstract

Phase change material (PCM)-based thermal energy storage units (TESU) have very low thermal conductivity that compromise their charging and discharging rate. The present study focuses on an enhancement in charging rate as well as an increase in the uniformity of the melting rate. A rectangular cavity consisting of two horizontal partial fins is studied. The horizontal partial fins are placed symmetrically in a PCM-based TESU. In the current work, the melting rate of PCM was enhanced using asymmetric arrangement while keeping all other parameters the same, thus showing the positive effect of asymmetric configuration in such storage systems. The position and the pitch of each fin is optimized to improve heat transfer characteristics of the TESU. The numerical investigation of the problem is performed. TESU with asymmetrically placed fins show better performance in terms of higher charging rate as well as uniformity of the charging rate. The asymmetric placement of the fins suggested by present study increased the charging rate by 74.3% on average as compared to the symmetrically placed fins in the storage system. The charging rate uniformity is improved by 43.7%. The asymmetric fin’s placement conserved the convection strength for a longer melting duration and so increased the Nusselt number by 80.2% as compared to the symmetrically placed fins. Thus, it can be concluded that the performance of asymmetric fins is better in the charging of PCMs than the symmetrically placed fins in a PCM-based TESU.

## 1. Introduction

Constant energy supply is an important aspect of any power generation system. Fluctuation in the supply of renewable energy makes it hard to implement into existing power systems. This fluctuation in supply can be minimized by storing excess amounts of renewable energy into some kind of thermal storage unit and then utilize it when demand rises. Nowadays researchers have been working on PCM-based energy storage systems to obtain the desired outcome. Phase change materials have high latent heat, thus making them an attractive choice for thermal energy storage units. However, these energy materials have very low thermal conductivity, which makes heat transfer to and from the TESU a challenging task. There are different techniques which have been used to improve the heat transfer characteristics of the energy storage systems. These techniques include microencapsulation of PCM [1], adding nanoparticles [2,3,4], absorption of phase change materials in metal foam [5,6,7,8,9,10,11,12,13,14] and insertion of fins [15,16,17,18,19,20].

Researcher have studied different geometrical shapes to enhance the thermal performance of the latent thermal energy storage system (TESS) [1,21,22]. Shendao Zhang et al. [1] prepared novel microencapsulated PCM to overcome the low thermal conductivity issue. The thermal performance of the double shell microencapsulation of the PCM promised its good application in thermal management and heat storage. Zhen Qin et al. [21] investigated melting behavior of PCM in a heat storage container. The results revealed that the geometry of the container affects the melting behavior of the PCM by influencing the natural convection. This change in shape positively impacts the surface contact area for both the source fluid and the PCM, thereby enhancing its charging and discharging rate. Bingkun Huang et al. [22] optimized the shape of the container to overcome the disadvantage of an extremely low melting rate for the remaining PCM at the bottom of the container. New designs for the PCM container are proposed to achieve a better melting rate by strengthening convection currents.

Different studies have been performed to observe the impact of container tilt angle on the performance of PCM-based TESU [23,24,25,26]. Babak Kamkari et al. [23] investigated the impact of different inclination angles of the PCM container on the melting behavior of the PCM. It was observed that decreasing inclination angle from 90° to 0° enhanced the convection currents. Jingde Zhao et al. [24] studied the impact of the rectangular cavity’s contact angle on the contact melting. The highest average melting rate was found for a tilt angle of 60°. Idris Al Siyabi et al. [25] performed numerical and experimental investigations on the impact of variation in the inclination angle of cylindrical TESS. Hot fluid was passed through the concentric copper tube inside TESS. The maximum melting rate was observed for inclination angle of 45°. Yuxuan Deng et al. [26] studied the thermal performance of erythritol-based LHTES utilizing solar heat.

One of the techniques used to enhance thermal conductivity of the PCM is to insert fins in the system [27,28,29,30,31,32]. This can further be classified into two groups: partition and partial fins. M. Gharebaghi et al. [27] studied the thermal performance of a TESU after the insertion of partition fins. The fins partitioned the PCM into sub-compartments called modules. The geometrical shape and temperatures of the module walls influence the charging and discharging rates of the TESU. M.J. Huang et al. [28] partitioned the PCM by inserting aluminum fins to pacify the temperature rise of the PV module. Mustafa S. Mahdi et al. [29] investigated the impact of partitioning the PCM-based storage system into small subparts. Wood flanges were used to partition the heat storage unit into two parts. The division of the LHSU into subparts enhanced the melting rate of the PCM by creating multiple melting fronts. S.F. Hosseinizadeh et al. [30] experimentally and numerically studied the impact of the insertion of partitioning metal fins on the thermal performance of a PCM-based heat sink. Anna Dmitruk et al. [31] fabricated aluminum inserts and studied their impact on the thermal performance of the charging and discharging of the PCM. Mohammad Ghalambaz et al. [32] applied Taguchi method to optimally place tubes in the latent heat storage system. The study suggested placement of one tube at the bottom.

Babak Kamkari et al. [33] studied the impact of partial fins on the melting behavior of PCM and the thermal performance of the LHSU for different temperatures of the hot wall. An increase in the number of partial fins improved the melting rate and the fin’s effectiveness, but this decreased with increase in temperatures of the wall. R. Akhilesh et al. [20] proposed novel design guidelines for a PCM-based heat sink using vertical fins as thermal conductivity enhancers. Nourouddin Sharifi et al. [17] reported the impact of fin numbers and length and the wall temperature on the melting of PCM. Chenzhen Ji et al. [34] studied the impact of the fin’s tilt angle on the heat transfer characteristics of PCM-based TESS. It was concluded that fastest the melting rate was achieved for fins with a downward tilt angle of −15°.

Most of the research related to optimization of the number of fins is performed for vertical partial and partitioned fins implemented in both heat storage systems and heat sinks to improve the thermal management of these systems [15,27,30,35]. In the case of horizontal partial fins, extensive studies have been done on placing the partial fins symmetrically in the PCM-based TESU. However, it has been observed that the melting rate becomes extremely compromised for the melting of the remaining PCM at the bottom. The fin’s contribution in melting rate is mainly in melting the PCM above the fin’s surface. The fin strengthens convection until all the PCM above its surface is melted. Therefore, the behavior of the PCM in vertical thermal energy storage units (TESU) is not linear with respect to time and there is great non-uniformity in the charging and discharging rates of the TESU.

The focus of the present study is to investigate the optimized position and pitch for a two-fins-based rectangular enclosure which results in a higher and uniform charging rate. Nonlinear melting behavior of the symmetrically placed fins-based system is studied. Initially, the melting rate is very high but in the later stages, the melting rate becomes extremely low. In order to overcome this issue, the fins’ positions are varied asymmetrically to increase the charging rate, making it persistently close to the constant average melting rate.

## 2. Materials and Methods

### 2.1. Physical Domain and Boundary Conditions

**Figure 1:**
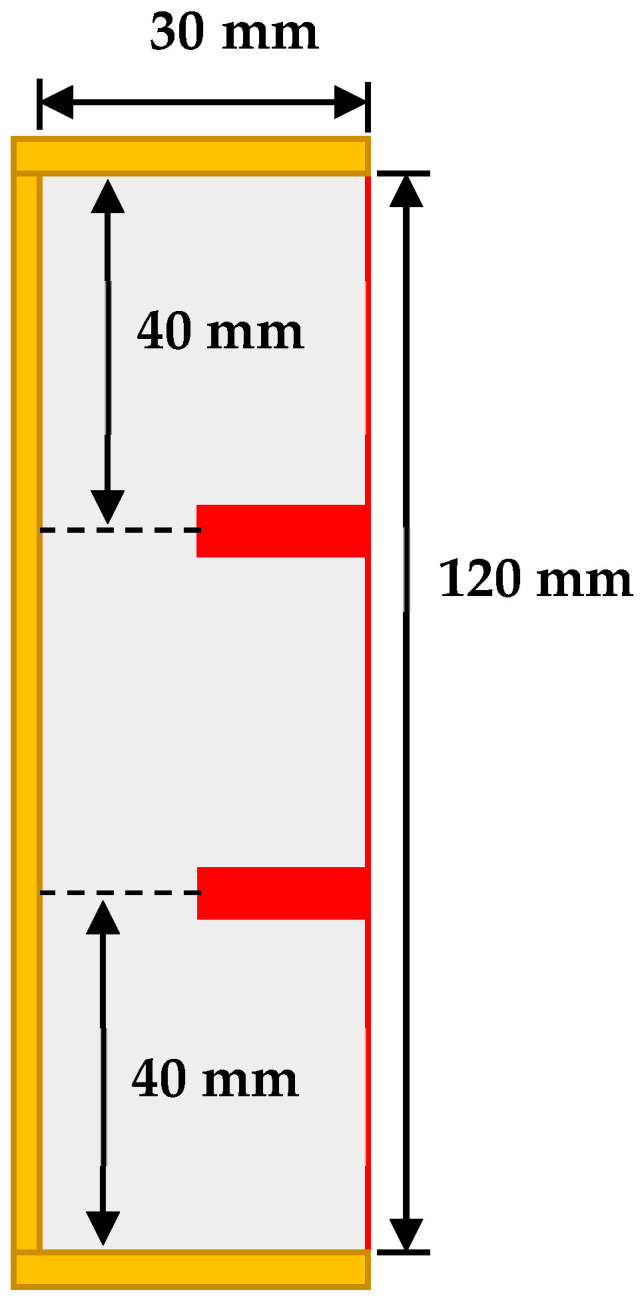
Rectangular cavity with 2 symmetrically placed fins.

A cavity of 30 mm × 120 mm was simulated to perform the analysis. Two horizontal fins each of 4 mm × 15 mm sizes were placed to see the effect of multiple fin positions on the melt rate of PCM. Figure 1 shows the symmetric fins position in the cavity along with the dimensions. The initial temperature of PCM zone is 26 °C, which is heated by the hot wall maintained at a temperature of 70 °C while all other sides were insulated. Lauric acid was used as the PCM; its thermophysical properties are listed in Table 1 [36].

**Figure 2:**
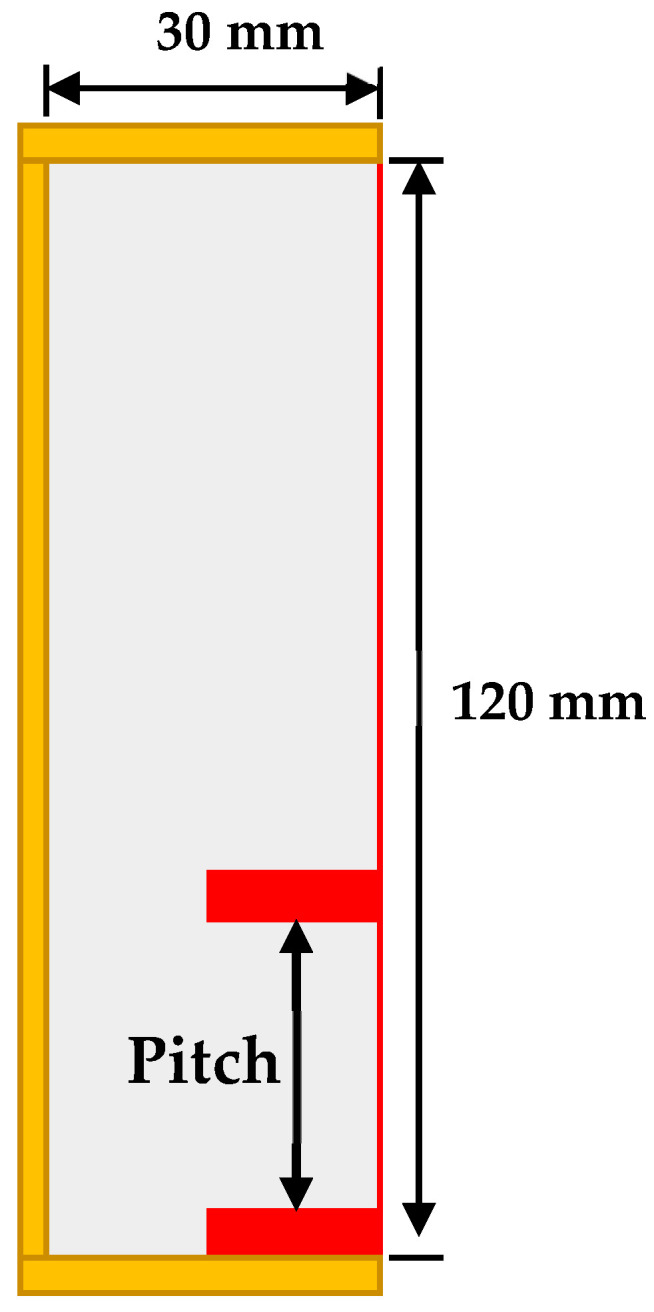
Rectangular cavity with 2 asymmetrically placed fins. The pitch was varied from 8 to 54 mm.

Conventionally, the fins are placed symmetrically for better distribution of heat across the PCM, as shown in Figure 1. This domain is simulated, so as to use its result as a reference for comparison with the proposed new fin’s placement methodology. The modified physical domain has two fins of the same size (4 mm × 15 mm) as in the symmetric case, but one fin is always at the bottom and the pitch and position of the 2nd fin is varied, allowing us to numerically investigate the problem. The physical domain of the modified configuration with asymmetrically placed fins is shown in Figure 2.

### 2.2. Numerical Model

ANSYS Fluent 19.2^®^ was used to solve the numerical domain of the problem. Enthalpy-porosity method along with a fixed domain was implemented to see the effect of fin position on the charging rate of lauric acid. Continuity, momentum, and energy equations are solved using coupled formulation to estimate the temperature distribution and melt fraction of PCM. Density was assumed to be constant and Boussinesq approximation was utilized to predict the melting time, heat flow rate and overall charging of the phase change material-based storage system [29]. The energy, momentum and continuity equations are as follows [37]:

(1) $$\frac{\partial u}{\partial x}+\frac{\partial v}{\partial y}=0,$$

(2) $$\rho \frac{Du}{Dt}+S\left(T\right)\cdot u=-\frac{\partial P}{\partial x}+2\frac{\partial }{\partial x}\left[\mu \frac{\partial u}{\partial x}\right]+\frac{\partial }{\partial y}[\mu \left(\frac{\partial u}{\partial y}+\frac{\partial v}{\partial x}\right)],$$

(3) $$\rho \frac{Dv}{Dt}+S\left(T\right)\cdot v=-\frac{\partial P}{\partial y}+2\frac{\partial }{\partial y}\left[\mu \frac{\partial v}{\partial y}\right]+\frac{\partial }{\partial x}[\mu \left(\frac{\partial u}{\partial y}+\frac{\partial v}{\partial x}\right)]+F_{B},$$

(4) $$\rho \frac{DH}{Dt}=\nabla \cdot (k\nabla T).$$

The model is simplified by the following assumption: the melted flow of PCM is laminar, incompressible and Newtonian, with no bulk solid sinking, negligible radiation heat transfers and no volume expansion. In Boussinesq approximation, density is assumed to be constant and the buoyancy term (Equation (5)) is incorporated in the momentum equation to account for the buoyancy driven flow as:

(5) $$F_{b}=g\overset{-}{\rho }\beta (T-T_{m}).$$

where *β* represents thermal expansion coefficient. The buoyancy incorporates the temperature difference across the PCM in the heat storage unit. In the energy equation, *H* is the total enthalpy of the PCM which is the sum of sensible and latent heat enthalpies.

(6) $$H=H_{s}+H_{l},$$

(7) $$H_{l}=Lf.$$

where *L* is latent heat of the PCM. The melting fraction (*f*) is defined in terms of the PCM temperature comparison to the solidus and liquidus temperatures:

(8) $$f=\left\{\begin{array}{cc} 0 & T<T_{s},\\ \frac{T-T_{s}}{T_{l}-T_{s}} & T_{s}<T<T_{l},\\ 1 & T>T_{l}. \end{array}\right.$$

The source term *S(T)* in the momentum equation accounts for the change in momentum of the melted PCM. The momentum of the melted PCM is dependent on the strength of the convection currents of the flow. The source term is

(9) $$S\left(T\right)=C_{mushy}\frac{{\left(1-f\right)}^{2}}{f^{2}+\epsilon }V_{i}.$$

$C_{mushy}$ is called the mushy zone constant. It accounts for the damping of the flow driven by natural convection, the higher the value, the more damping of the velocity. Therefore, the source term dampens the momentum of the flow and affects the melting behavior of the PCM. For the particular nature of the PCM melting problem, the mushy zone constant has to be tuned. In the current study, a mushy zone constant of 10^5^ is considered appropriate for the problem’s solution. The division by constant ‘*ε*’ of a very small value is done to avoid division by zero in the case of zero melting fraction.

The numerical investigations are performed by invoking continuity and momentum equation residuals of 10^−6^ as a convergence criterion. The energy equation residuals are set to be 10^−9^. The mesh independent study is conducted and no significant change in result is observed for mesh size smaller than 0.3 mm. The time step size for each simulation is set to be 0.1 s while the total number of iterations for each step is 200. The numerical results of the cavities with no and a single fin are compared with experimental data for the model validation. The same model is utilized to obtain all the results of the two-fin configuration (both symmetric and asymmetric).

### 2.3. Numerical Model Validation

**Figure 3:**
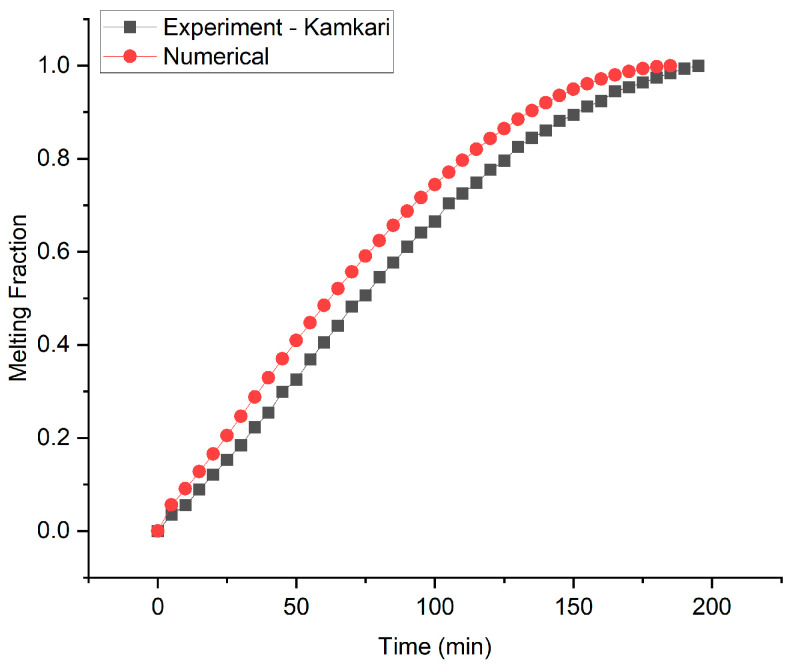
Numerical results comparison with experimental results reported by Kamkari.

The numerical model is validated by comparing numerical results with experimental results reported by Kamkari [36]. The numerical results were produced by simulating the melting behavior of PCM in a rectangular cavity with one vertical wall kept at 70 °C while all the other walls are insulated. The rectangular enclosure has 50 mm of width, 50 mm of depth and 120 mm of height. Lauric acid is used as the PCM for heat storage. The results comparison is shown in Figure 3. The results show good agreement with an average error of 7.58%.

**Figure 4:**
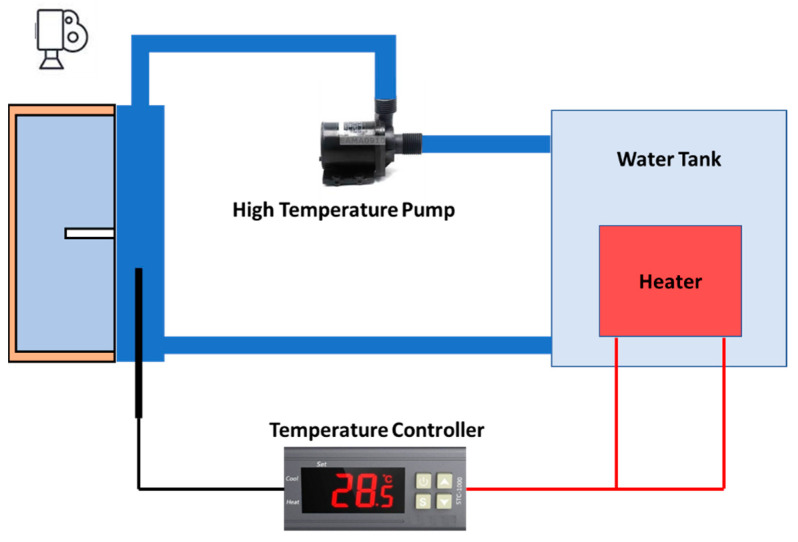
Experimental setup.

To further validate the model, an experiment with a single fin cavity is performed. The experimental setup is shown in Figure 4. The experimental setup can be divided into three sections, the test section, the heat transfer fluid section, and the data acquisition section. In the test section, a rectangular cavity is filled with melted Lauric acid. The hot wall is made of Aluminum and the other walls are made of acrylic. The whole cavity is insulated with 50 mm thick Styrofoam sheet to make sure no heat transfer occurs from the acrylic walls. A hot water jacket is attached to the hot wall to maintain its temperature at 70 °C.

The heat transfer fluid section consists of constant temperature hot and cold reservoirs, implemented with a temperature controlling unit. The temperature controlling unit consists of a temperature controller, temperature feedback via thermocouples, and a 2000 W heater. A PID based temperature controller integrated with a K-type thermocouple is used for temperature regulation.

The water from the cold reservoir is circulated through a jacket attached to the aluminum wall for 12 h to make sure there is a uniform temperature of 25 °C throughout the PCM. Before carrying out the experiment, the hot water temperature is raised to 70 °C. Initially, the experiment’s cold water is stopped and water from the hot reservoir is pumped through the water jacket. Initially, the temperature dropped down a few degrees below 70 °C, because of the hot water mixing with the cold fluid remaining in the pipes and water jacket. Through the feedback system, the PID controller heats the hot water reservoir to raise and maintain the aluminum wall temperature at 70 °C. The heating of the hot water to 70 °C before the start of the experiment gives refined results with better agreement with the numerical results. Otherwise, when starting the experiment without prior heating of the water, the PID controller takes a few minutes to raise the temperature of the wall to the required 70 °C. This compromises the results and offers more disagreement between the experimental and numerical results. A camera has been used to capture images of the PCM through the transparent acrylic walls. Solid and liquid PCM regions are distinctly visualized. Lauric acid becomes transparent after melting, while solid PCM is whitish in appearance. Coral draw is used to calculate the temporal evolution of the respective areas of the liquid and solid PCM regions. The melting fraction is the ratio of melted PCM region area to the remaining solid PCM area. The experiment was performed thrice to verify the repeatability of the experiment. 

**Figure 5:**
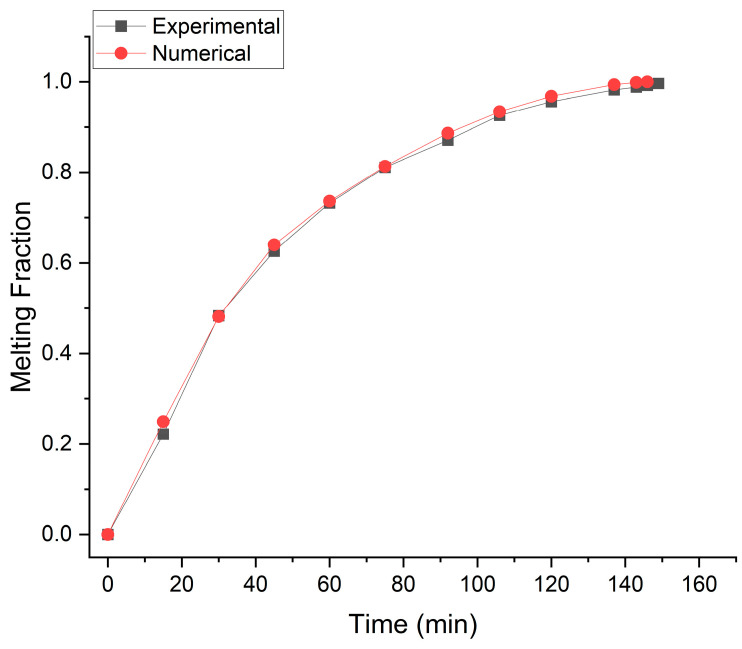
Numerical and experimental results comparison for fin placed at the mid position.

The same physical domain is simulated in Ansys Fluent to validate the numerical model results with the experimental results described above. The comparison is shown in Figure 5. The results show good agreement between numerical and experimental data with an average error of 2.07%.

## 3. Results and Discussion

### 3.1. Melting Rate

**Figure 6:**
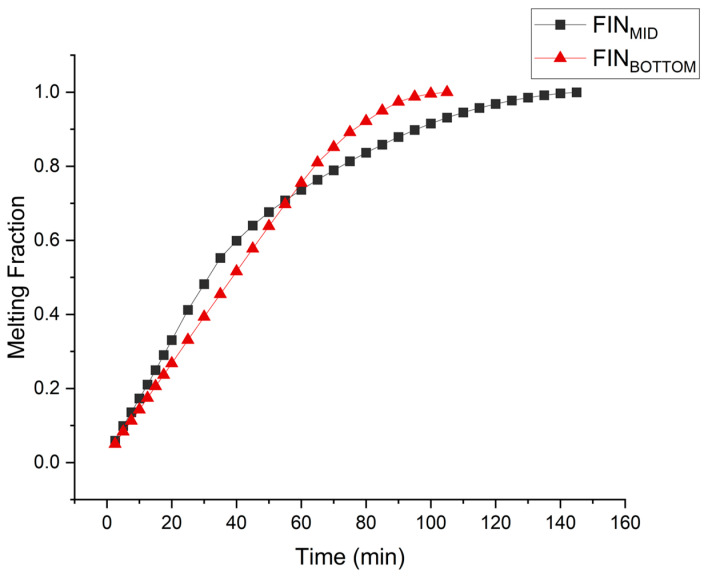
Numerical results comparison for fins placed at the mid and bottom positions.

The melting behavior of PCM in the rectangular cavity is nonlinear as depicted in Figure 5. In the symmetric fin configuration, the PCM at the bottom of the cavity melts at a very low rate, thus negatively affecting the overall charging rate. Therefore, the fins are lowered to achieve better melting rate uniformity and reduction in complete melting time. Conventionally, in the case of a single fin cavity, keeping in view the symmetric arrangement, the fin is placed at the mid position. In order to see the effect of a single fin asymmetric position, simulations of a fin placed at the bottom are performed and compared with the symmetric fin results as shown in Figure 6. The result shows drastic improvement in the charging rate when the single fin is placed at the bottom position. Initially the melting rate, which is directly related to the charging rate, compromises in case of the asymmetric placement of the fin. However, the melting rate compromise being delayed near to the very completion of melting process overcomes this disadvantage. The overall charging time for symmetric placement of the fin was 147.2 min which is reduced to 106.5 min in the case of fin placed at the bottom position. The overall charging rate was improved by 27.6%.

The same phenomenon is utilized in two-fin configurations in which one fin is placed at the bottom and the position of the second fin is varied. In order to find the optimized position of second fin, the pitch is incrementally varied from 8 to 54 mm. Numerical investigations are carried out for the cavity (width 30 mm, depth 50 mm and height 120 mm) to investigate the optimum position for the second fin. 

**Figure 7:**
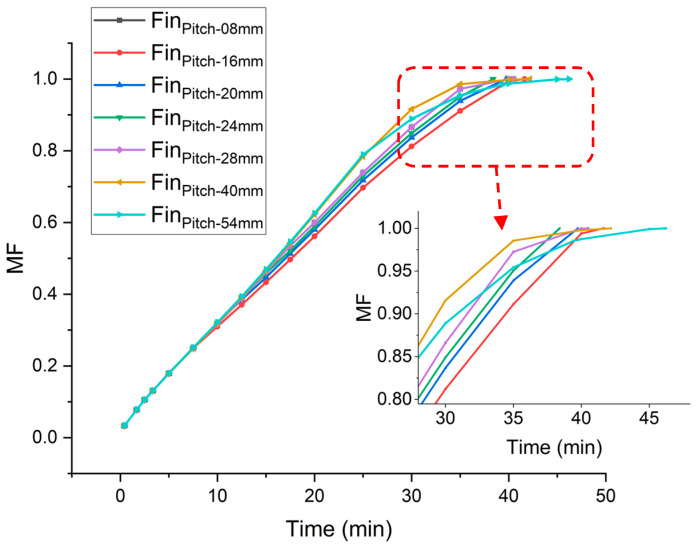
Time vs Melt Fraction at different pitch.

It is observed that the fin’s placement at a higher pitch/position initially leads to higher melting rate, but at later stages of melting the melting rate is extremely reduced. Lowering the second fin towards the bottom compromises the melting rate at the start of melting process. At a very high position, there is an extreme compromise at later stages of the melting process, and at a very low position, there is an appreciable compromise in the melting rate from the very start. Therefore, there exists an optimum position/pitch at which the overall melting rate is highest. As Figure 7 depicts, at a pitch of 24 mm the second fin’s placement gives optimized results in terms of overall melting rate and reduced complete melting time. 

**Figure 8:**
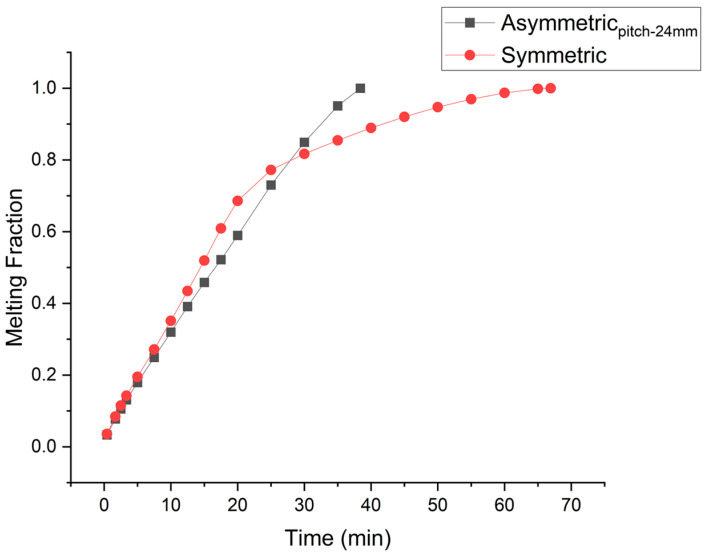
Time vs melt fraction for two fin symmetric and asymmetric (pitch-24 mm).

**Figure 9:**
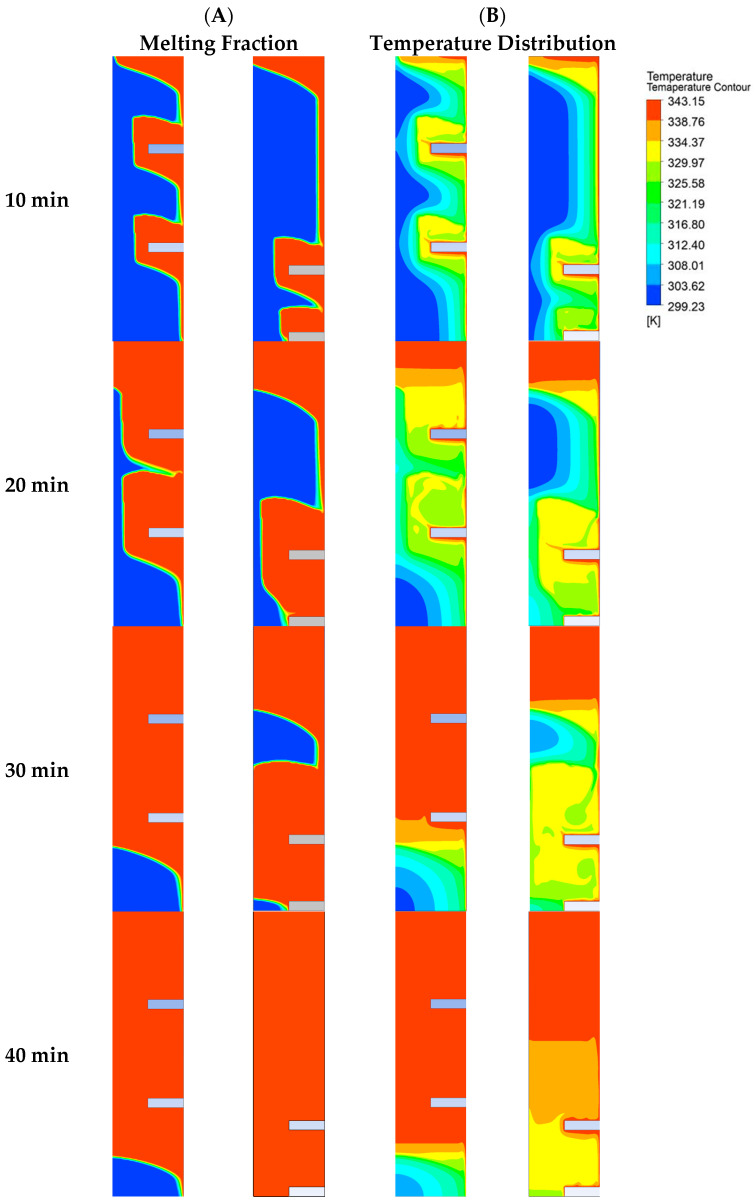
(**A**) Temporal evolution of melting front, (**B**) temperature distribution during melting process (**left**; symmetric, **right**; asymmetric at pitch of 24 mm).

Such melting behavior can be explained by exploring the overall melting regime. At the start of melting, a high temperature difference between the hot plate and the solid is responsible for the higher rate of heat conduction. Symmetric placement of the fin favors conduction, which is responsible for the initial higher melting rate. Therefore, as the second fin is placed at the mid position of the cavity, the melting rate is initially higher. But as the melted PCM layer between the hot plate and solid PCM widens, thermal resistance to heat conduction increases. This widened gap favors the convection flow. Now convection currents can overcome the viscous stresses. The fin’s contribution in convection driven PCM melting is mainly in the melting of the solid PCM above the fin. There is minor contribution of the fin in melting of the PCM below the Fin’s position. So as the PCM is melted above the fin, convection starts decaying and the melting rate becomes extremely compromised. Comparison of the temporal melting rate for the asymmetric fin’s placement with the conventional symmetric placement of the fins is depicted in Figure 8. It is evident in the case of symmetrically placed fins that after a melt fraction of 0.69, the melting rate becomes extremely compromised. On the other hand, in the case of the Asymmetric fin, the fin being placed at the bottom delays such compromise till the completion of the melting process. The comparison of melting fractions in Figure 9A shows that initially the melting rate in the case of symmetric fins is higher. The orange color shows melted PCM and the blue color represents the solid PCM. Analyzing melting front evolution (at 10 min) shows that the solid PCM above the fin’s surface melts fast as compared to the PCM below the fin’s surface. Therefore, there is a bigger gap between the solid PCM and the fin’s surface above the fin. This represents the dominance of convection that is more affective above the fin surface. The temperature distribution shown in Figure 9B illustrate that after the melting of PCM above the fin, density difference-driven convection flow extremely damps down the cause of disappearance of temperature difference in the top part of the cavity. In the case of symmetric fins, especially when the number of fins is lower, thermal stratification is prominent. However, in the case of the asymmetric fin’s placement, moving the fin to the bottom enhances density driven flow, reduces thermal stratification, and so increases the temperature uniformity. 

### 3.2. Nusselt Number

**Figure 10:**
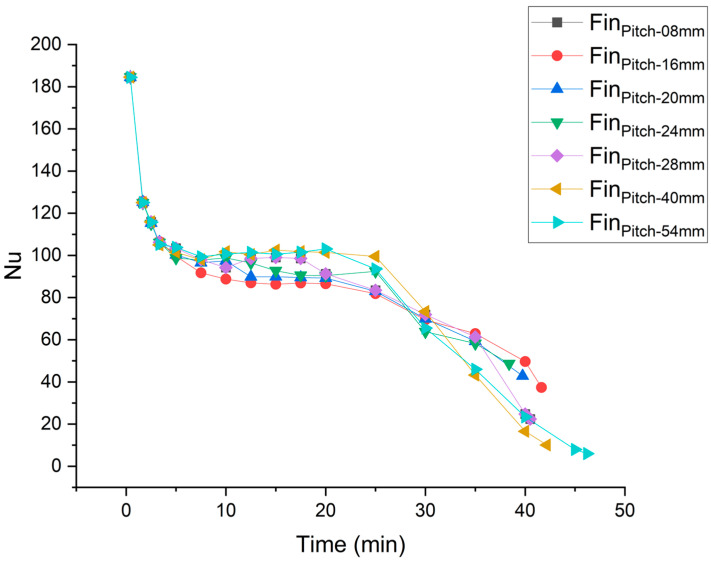
Nusselt number variation with time at different pitches.

**Figure 11:**
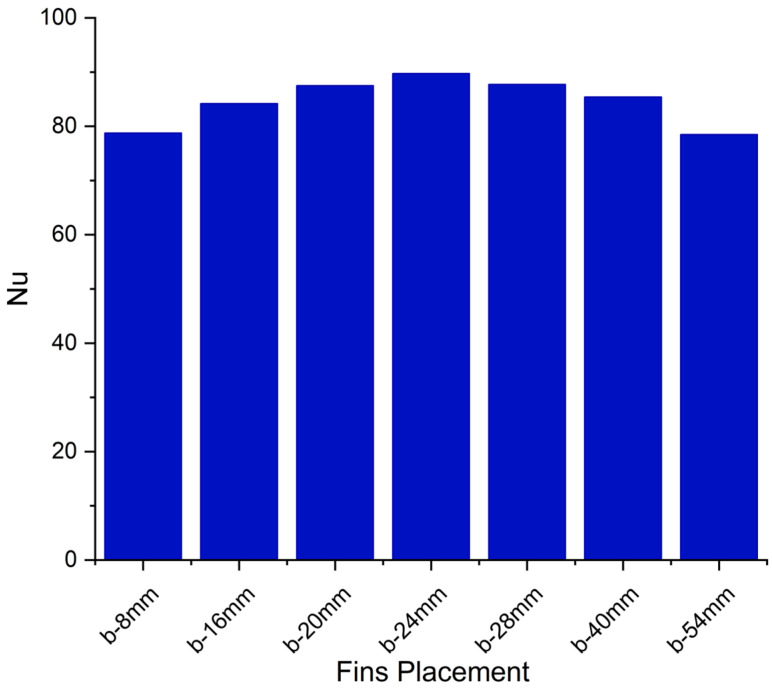
Average Nusselt number at different pitches.

Nusselt numbers for different positions of second fin are depicted in Figure 10. Overall, the depiction of Nusselt number exhibits three distinct regimes of PCM melting. At the start of the melting, there is a very high Nusselt number, but it is drastically reduced shortly thereafter. This is because of high heat conduction at the start of melting, driven by higher temperature difference between the hot wall and solid PCM. However, as the melted PCM layer between the hot wall and solid PCM widens, conduction becomes sharply compromised. Now, as more PCM is melted, the gap between the hot wall and solid PCM widens, and convection currents can now overcome the viscous forces. This convection dominance is responsible for not letting the Nusselt number decrease further. Then, there is the third regime where the Nusselt number drops further till the melting of all the PCM. This drop in the Nusselt number represents weakening of the convection heat transfer. Figure 10 depicts the impact of the second fin’s position on the Nusselt number. As the fin is lowered, it prolongs the convection dominance regime, increasing the overall heat transfer rate and reducing complete melting time. However, placement of the fin near the bottom fin prolongs the convection dominance region but compromises the strength of the convection. Therefore, it decreases the Nusselt number in the convection dominance regime and compromises the initial melting rate. The average Nusselt number is plotted for different fin positions of the second fin in Figure 11. Moving the second fin from bottom to top increases the average Nusselt number, up till a pitch of 24 mm. Afterwards the average Nusselt number decreases with an increase in the pitch until the top. It is observed that the Nusselt number compromise per unit variation in the pitch is more pronounced if the fin is moved below the optimum pitch as compared to moving the fin upward. The optimum overall Nusselt number is achieved at a pitch of 24 mm. 

**Figure 12:**
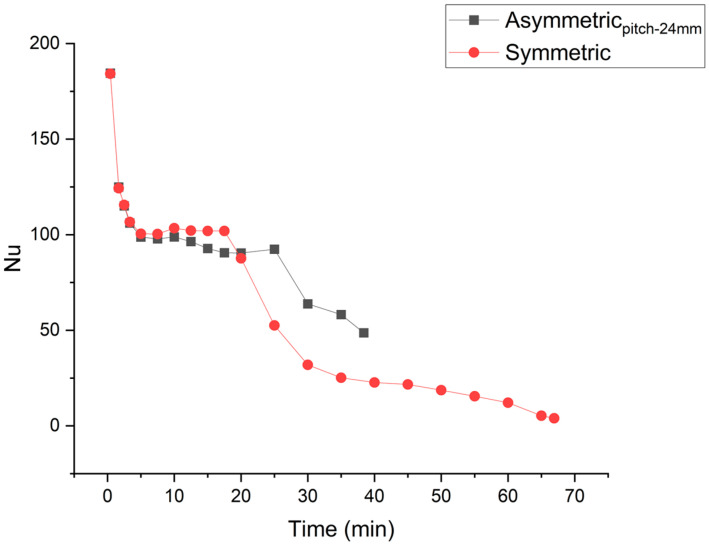
Nusselt number for two fin symmetric and asymmetric configurations (pitch-24 mm).

The asymmetric fin’s placement optimizes the initial higher Nusselt number, average Nusselt number in convection dominance regime, and prolongation of the convection dominance regime. Nusselt numbers for asymmetric (optimized) and symmetric fin placements are compared in Figure 12. Asymmetrically placed fins show prolongation of the convection dominance regime. Convection weakens very late in the melting process. In the case of symmetric placement of fins, an early decay in convection compromises the Nusselt number and delays the completion of the melting process. 

**Figure 13:**
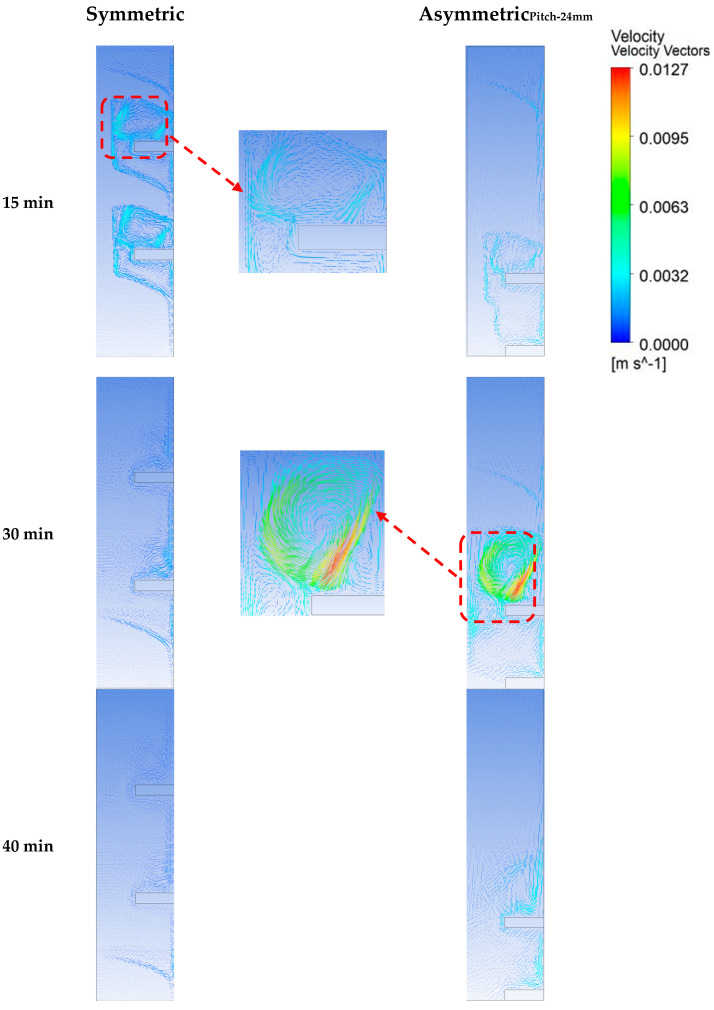
Velocity vectors for symmetric and asymmetric fin configurations (pitch −24 mm).

The velocity vectors plotted in Figure 13 depict the evolution of buoyancy-driven flow. For the symmetric case (10 min), the convection flow initially has higher velocity, which strengthens the convection currents and so leads to higher melting rate as compared to the asymmetric fins. However, with time the velocity of flow damps down. This is when most of the PCM is melted above the fins. In the case of asymmetric fins, the convection currents keep their strength, except at the very end of melting process. Therefore, in the case of asymmetric fins, such behavior of the flow, which retains appreciable velocity over a longer melting range, represents the prolongation of the convection dominance regime represented by plot of Nusselt numbers in Figure 12.

### 3.3. Thermal Energy Stored

**Figure 14:**
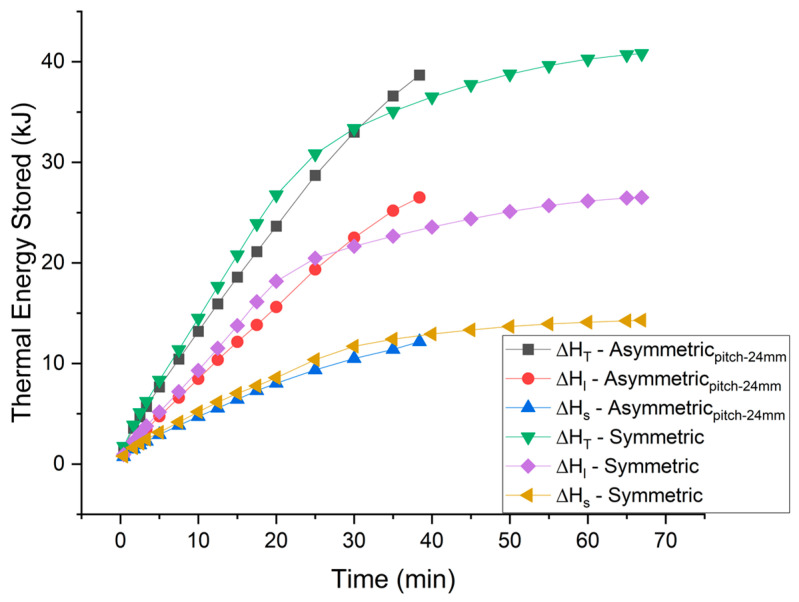
Thermal energy storage for symmetric and asymmetric fin configurations (pitch-24 mm).

Temporal thermal charging of the TESU is divided into sensible and latent heat storages, shown in Figure 14, for both symmetric and asymmetric arrangements of the fins. Latent heat storage rate is exactly the same as the melting rate. Therefore, in the case of asymmetric fins, the latent heat storage rate initially compromised but stayed near to the average value. However, in the case of symmetric fins, the latent heat storage rate was extremely compromised in the later stages of melting. Sensible heat storage throughout the melting process stays lower for asymmetric fins. In the asymmetric fin’s placement, the sensible heat storage distribution across the TESU becomes compromised because both the fins are located near the bottom and away from the PCM in the upper part of the TESU. This compromise in sensible heat storage rate causes a short delay in the time, during which total heat stored in the case of asymmetric fins is higher than in the case of symmetric fins. 

### 3.4. Melting Rate Uniformity

To get an insight into melting rate uniformity, the whole melting process is divided into 10 equal melting ranges from 0 to 1 with a difference of 0.1 melting fraction. (0–0.1, 0.1–0.2, 0.2–0.3, 0.3–0.4, 0.4–0.5, 0.5–0.6, 0.6–0.7, 0.7–0.8, 0.9–1). Local mean melting rate ${\bar{MR}}_{Ri}$ and overall melting rate ${\bar{MR}}_{avg}$ for complete melting are used to calculate the standard deviation of the specific local melting range by using Equation (11).

(10) $${\bar{MR}}_{avg}=\frac{1}{CMP},$$

(11) $${SD}_{MFR-Ri}=\sqrt{\frac{1}{10}\sum_{i=1}^{10}\frac{{\left({MR}_{Ri}-{\bar{MR}}_{avg}\right)}^{2}}{10}}.$$

**Figure 15:**
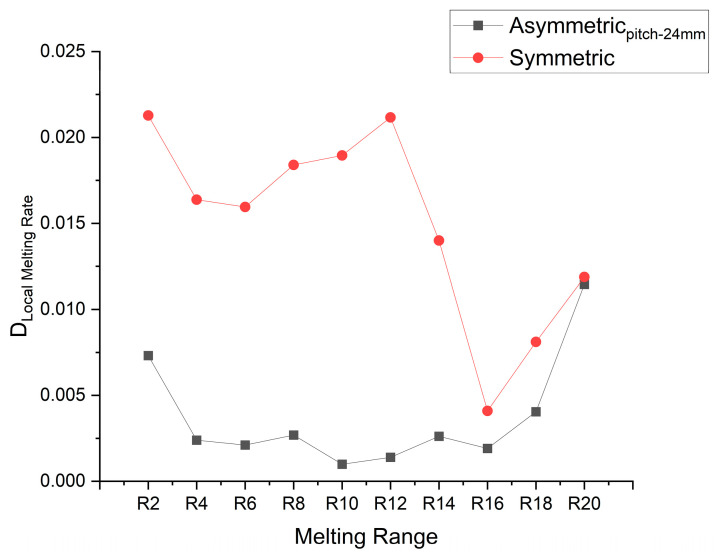
Deviation/difference of local averaged melting rate from the overall mean melting rate.

The deviation of the local mean melting rate from the overall average melting rate is plotted in Figure 15. In the case of symmetrically placed fins, the local mean melting rate is initially very high, then later the rate becomes extremely compromised. However, in the case of the asymmetric fin’s placement, the local mean melting rate is compromised late and relatively less, as compared to the asymmetric arrangement. By lowering the fin to the bottom position, melting fraction uniformity improved by 43.7%.

## 4. Conclusions

The study focuses on the improvement of melting rate uniformity, increasing melting rate, and so also the charging rate of the PCM based TESU. Conventionally the fins are placed symmetrically, dividing the PCM cavity into equal parts. However, the nonlinear melting behavior of PCM does not favor the arrangement. The placement of the fin affects buoyancy force-driven natural convection flow. The buoyancy force driven by the temperaturedifferences is dependent on the position of the fin with respect to the PCM. The present study numerically investigates how the fin’s position can exploit the melting behavior of the PCM to improve the thermal performance of the storage system. The fins are placed asymmetrically with one fin at the bottom of the enclosure, while the position of the second fin is varied to find the optimum pitch. At the optimum pitch, the charging rate is at its maximum, and varying from this position negatively affects the melting rate. The higher pitch shortens the convection dominance regime and causes an extremely low melting rate at the later stage of PCM melting. However, too low a value of pitch compromises the average value of the Nusselt number, and lowers the strength of convection in the convection dominance region. Therefore, it leads to an overall lower charging rate in the earlier stages of melting. In the present study, the optimized pitch is found to be 24 mm. Results of the optimized fin’s arrangement are compared with the symmetrically placed storage system. The average melting rate and melting rate uniformity are improved by 74.3 and 43.7%, respectively. The average Nusselt number was increased by 80.2%, for asymmetric placements of the fin. It is suggested to further study the optimum position of multi-fin based TESU units having more than two fins. The impact of aspect ratio on the optimum position of the fins need to be further explored as well.

## Figures and Tables

**Table 1:** Thermophysical properties of Lauric Acid.

$T_{M}$	**316 K**
$\rho_{S}$	940 kg/m^3^
$\rho_{L}$	885 kg/m^3^
$C_{P,S}$	2,180 J/kg K
$C_{P,L}$	2,390 J/kg K
$k_{S}$	0.16 W/m K
$k_{L}$	0.14 W/m K
β	0.0008 K^−1^
H	187,200 J/kg

## Data Availability

All data is included in the manuscript.

## Nomenclature

T_M_	Melting temperature (K)
ρ_S_	Density of solid (kg/m^3^)
ρ_L_	Density of liquid (kg/m^3^)
C_P,S_	Specific heat capacity of solid PCM (J/kg·K)
C_P,L_	Specific heat capacity of liquid PCM (J/kg·K)
k_S_	Thermal conductivity of solid (W/m·K)
k_L_	Thermal conductivity of liquid (W/m·K)
β	Thermal Expansion Coefficient (K^−1^)
H	Total Enthalpy (J/kg)
H_L_	Latent Enthalpy (J/kg)
H_S_	Sensible Enthalpy (J/kg)
Nu	Nusselt Number
f	Melting Fraction
